# Supplementary material for: A LATS biosensor screen identifies VEGFR as a regulator of the Hippo pathway in angiogenesis
Source: Nat Commun. 2018 Mar 13;9:1061. doi: 10.1038/s41467-018-03278-w (PMC5849716; doi:10.1038/s41467-018-03278-w)

A LATS biosensor screen identifies VEGFR as a regulator of the Hippo pathway in angiogenesis

Azad et al.

Supplementary Information

Supplementary Figure 1

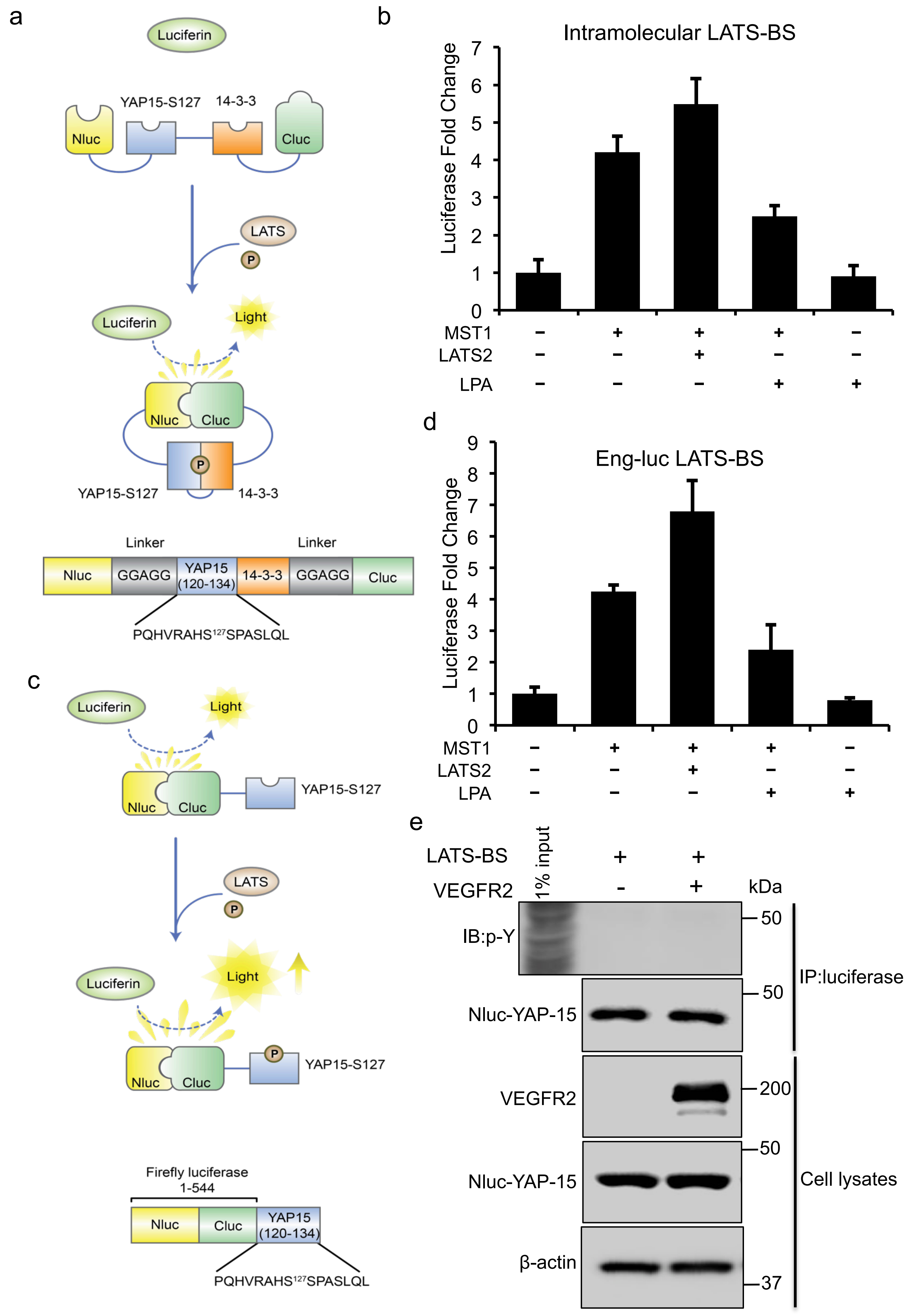

Supplementary Figure 1. Establishment of intramolecular and engineered luciferase LATS biosensors. (a) Domain structure and mechanism of action for an intramolecular LATS-BS. For intramolecular LATS biosensor, firefly luciferase amino acids 1-416 (N-luciferase, Nluc) were fused to the N-terminal of YAP15 (120-134) separated by a glycine/alanine linker (GGAGG). Within the same open reading frame, luciferase amino acids 394-550 (C-luciferase, Cluc) were fused to the C-terminal of 14-3-3 separated by a glycine/alanine linker. For this biosensor, LATS phosphorylates YAP15-S127 to cause a conformational change in the intramolecular LATS-BS, leading to luciferase complementation and detectable biosensor activity. (b) Validation of the intramolecular LATS-BS. The intramolecular LATS-BS was transfected alone or together with LATS2 or/and MST2 into HEK293 cells and biosensor activity was determined 48 hours after transfection by luciferase assay. For LPA treatment, cells were stimulated with 10 μM LPA for 1 hour before collection (n = 3). (c) Domain structure and mechanism of action for an engineered luciferase (Eng-luc) LATS-BS. Based on an approach previously published[1] , the terminal 7 amino acids from firefly luciferase were removed to create Eng-luc. This construct was fused to the N-terminal of YAP15 (120-134). This brings the luciferase site in close proximity to YAP15-S127 such that LATS-dependent phosphorylation of YAP-S127 modulates luciferase activity directly. (d) Validation of the Eng-luc LATS-BS. The Eng-luc LATS-BS was transfected alone or together with LATS2 or/and MST2 into HEK293 cells and biosensor activity was determined 48 hours after transfection by luciferase assay. For LPA treatment, cells were stimulated with 10 μM LPA for 1 hour before collection (n = 3). (e) The LATS-BS is not tyrosine-phosphorylated at baseline or with VEGFR2 overexpression. LATS-BS was expressed in HEK293 with or without VEGFR2 expression. LATS-BS was immunoprecipitated using an antibody against firefly luciferase and tyrosine phosphorylation was observed by western blot. Total cell lysate (“input”) was used as a positive control for tyrosine phosphorylation.

Data are represented as mean ± SD.

Supplementary Figure 2

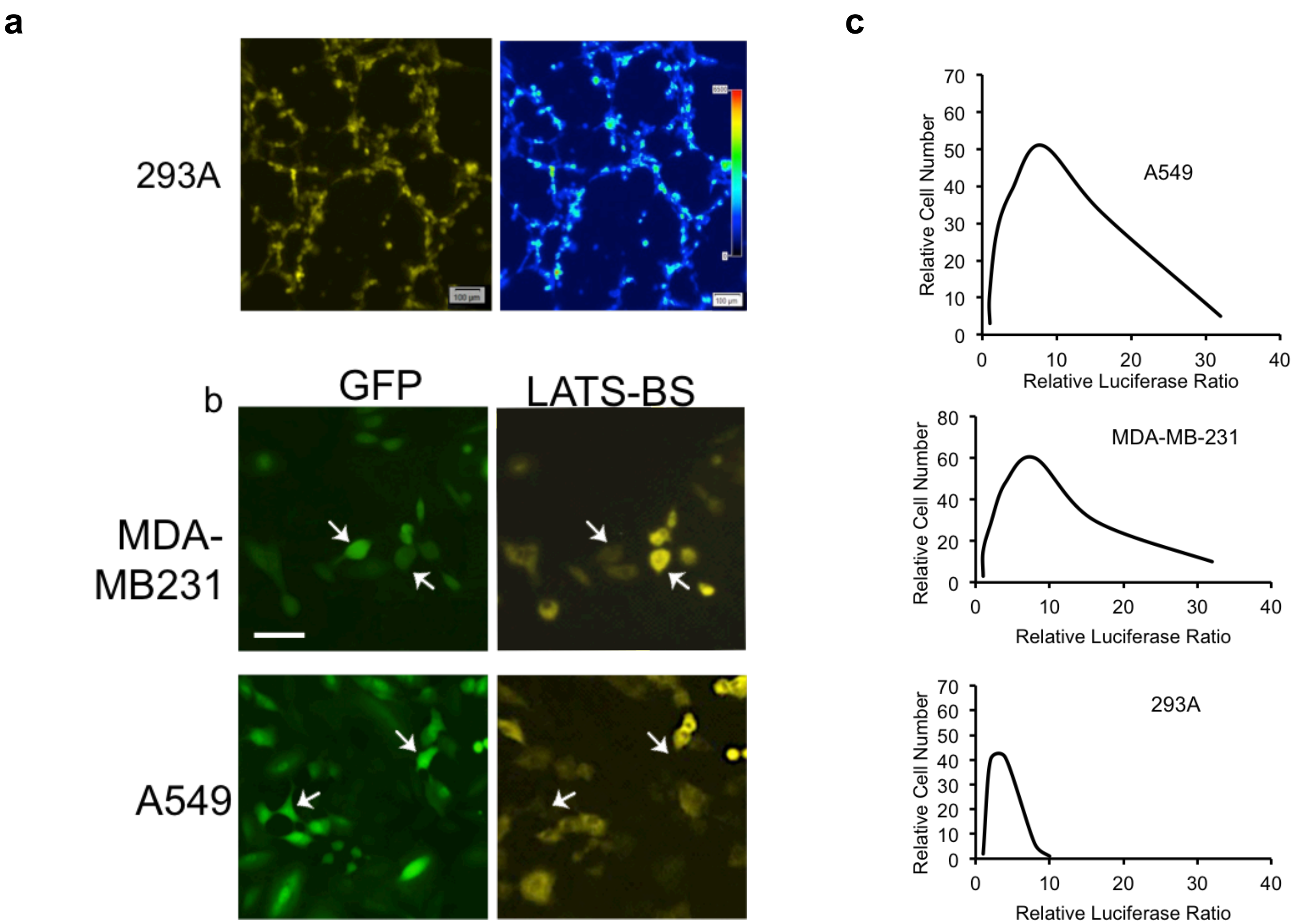

Supplementary Figure 2. Live cell monitoring of LATS activity using LV200 BLI system. (a) Representative images of LATS-BS activity in HEK293A cells captured using the LV200 BLI system after addition of 150 µg/mL D-luciferin (left). Heat map of LATS-BS intensity from same image (right). Scale bar denotes 100 µm. (b) LATS-BS activity is heterogeneous in cancer cell lines MDA-MB231 and A549**.** LATS-BS was stably overexpressed using a lentiviral vector containing GFP. Cells were imaged using LV200 BLI system for GFP and luciferase, independently. Notably, the levels of GFP and LATS-BS activity are not correlated in individual cells indicating that variation in LATS-BS activity is not due to differences in virus infection. Arrows show cells in which GFP expression is different from LATS-BS activity level. (c) Heterogeneity of endogenous LATS kinase activity in A549, MDA-MB231 and HEK293A cell lines. LATS-BS was stably overexpressed in each cell line and cells were imaged using the LV200 BLI system. Heat maps were generated for each image using CellSens software and distributions were created.

Supplementary Figure 3

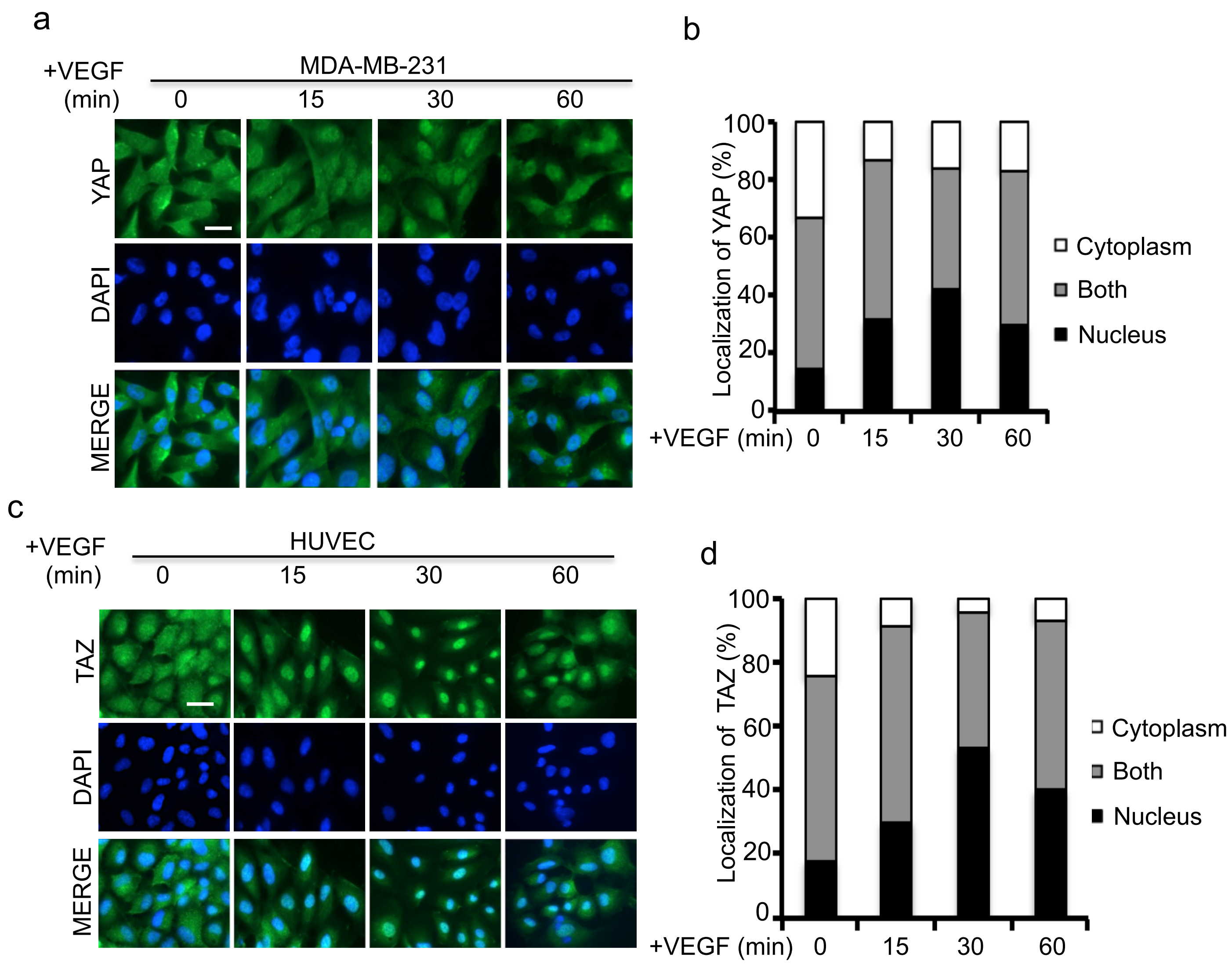

Supplementary Figure 3. VEGF treatment promotes YAP and TAZ nuclear translocation. (a,b) VEGF stimulation increases nuclear localization of YAP in MDA-MB231. (a) Representative images of YAP immunostaining after cell treatment with 100 ng mL^{-1} VEGF for the indicated times. Scale bar represents 15 μm. (b) Subcellular localization of YAP was quantified in at least 200 cells from the immunostaining. (c,d) VEGF stimulation increases nuclear localization of TAZ in HUVEC. (c) Representative images of TAZ immunostaining after cell treatment with 100 ng mL^{-1} VEGF for the indicated times. Scale bar represents 15 μm. (d) Subcellular localization of TAZ was quantified in at least 200 cells from the immunostaining.

Supplementary Figure 4

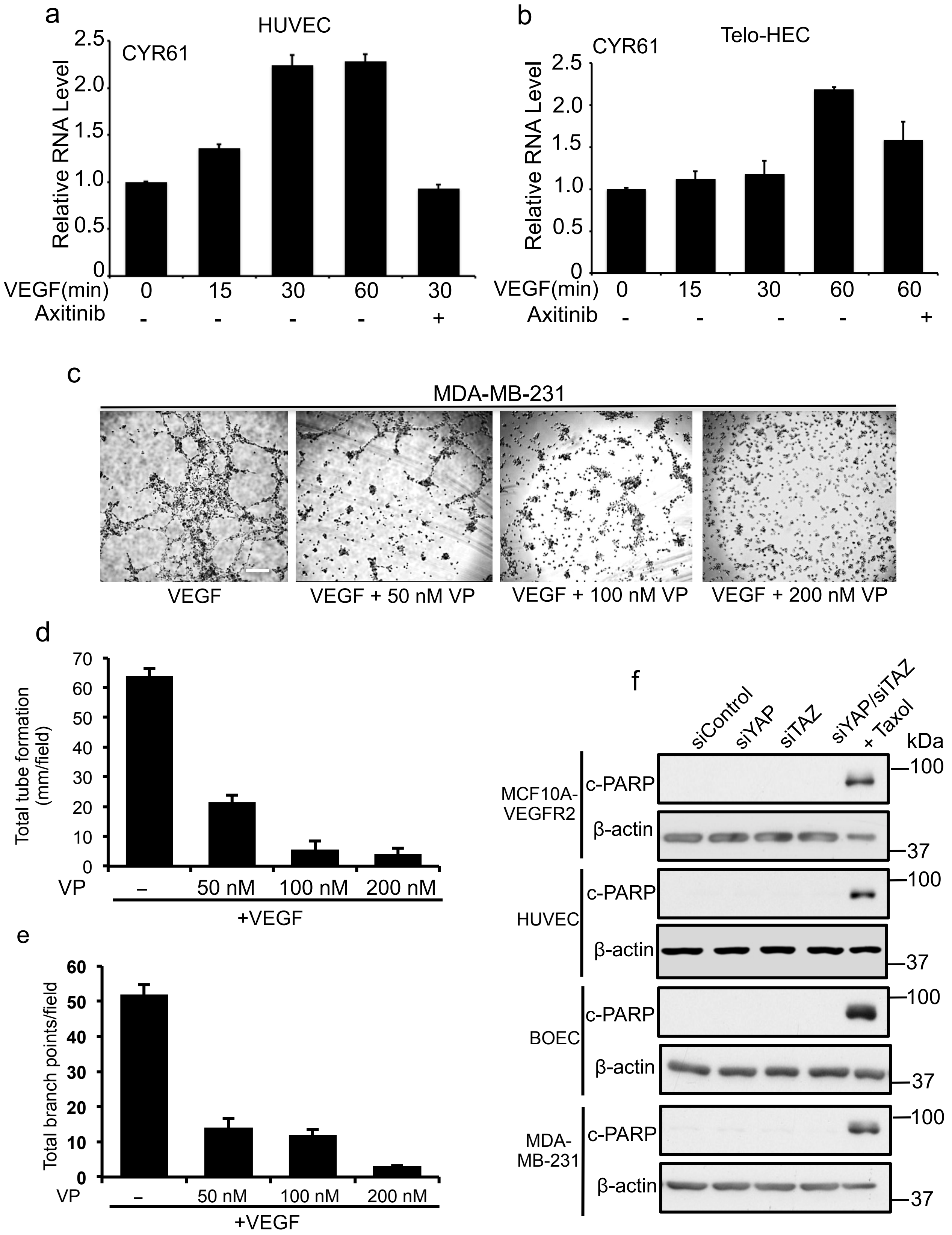

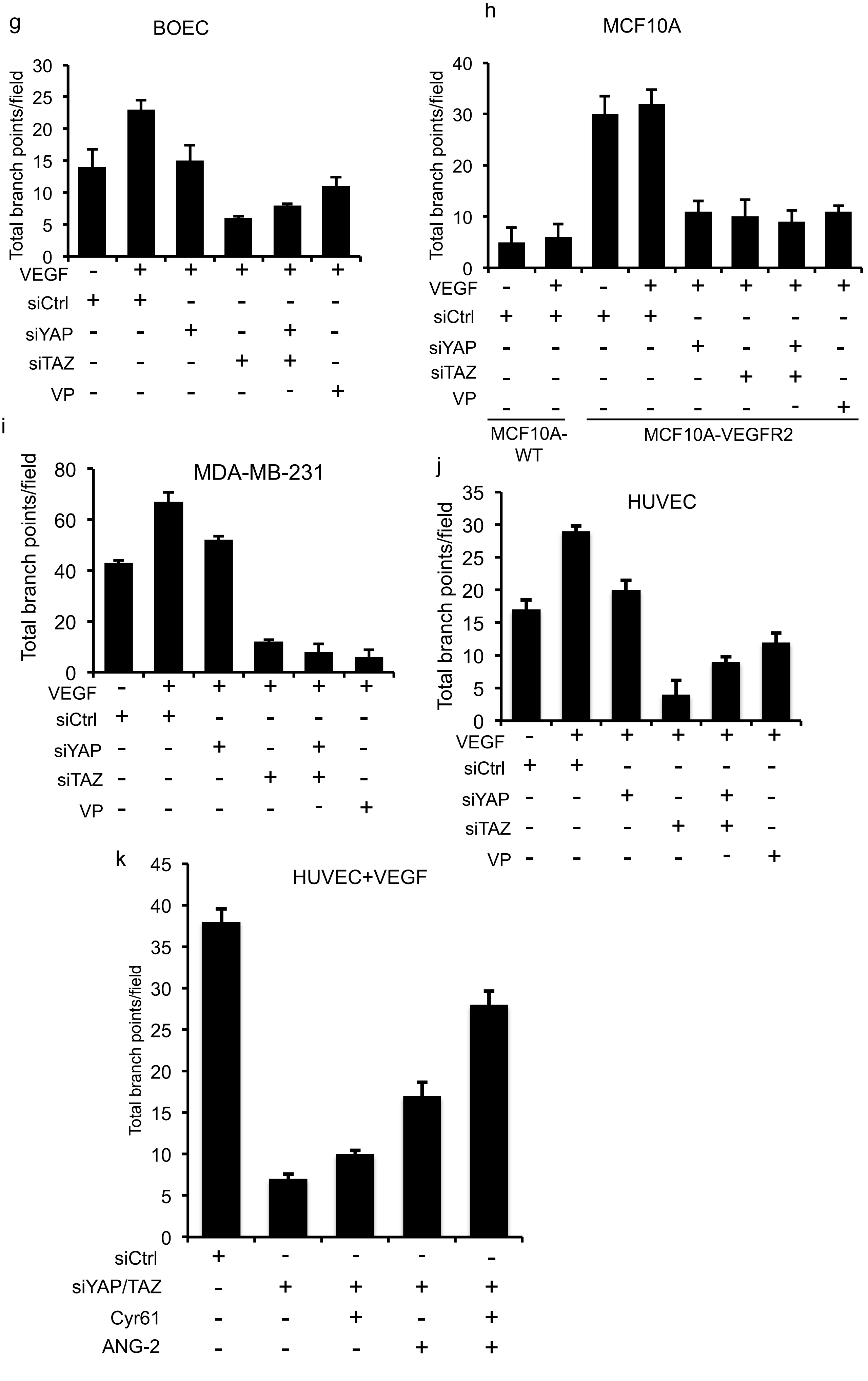
g
BOEC
Total branch points/field
0
5
10
15
20
25
30
VEGF - + + + + +
siCtrl + + - - - -
siYAP - - + - + -
siTAZ - - - + + -
VP - - - - - +
h
MCF10A
Total branch points/field
0
10
20
30
40
VEGF - + - + + + + +
siCtrl + + + + - - - -
siYAP - - - - + - + -
siTAZ - - - - - + + -
VP - - - - - - - +
MCF10A-WT
MCF10A-VEGFR2
i
MDA-MB-231
Total branch points/field
0
20
40
60
80
VEGF - + + + + +
siCtrl + + - - - -
siYAP - - + - + -
siTAZ - - - + + -
VP - - - - - +
j
HUVEC
Total branch points/field
0
5
10
15
20
25
30
35
VEGF - + + + + +
siCtrl + + - - - -
siYAP - - + - + -
siTAZ - - - + + -
VP - - - - - +
k
HUVEC+VEGF
Total branch points/field
0
5
10
15
20
25
30
35
40
45
siCtrl + - - - -
siYAP/TAZ - + + + +
Cyr61 - - + - +
ANG-2 - - - + +

Supplementary Figure 4. Functional interaction of VEGFR and the Hippo pathway in angiogenesis and VM. (a,b) VEGF stimulation increases YAP/TAZ transcriptional co-activation of *CYR61* in HUVEC (a) as well as in Telo-HEC (b). Cells were treated with 100 ng mL^{-1} VEGF for the indicated times. Total RNA was collected and *CYR61* expression was measured by qRT-PCR. For some samples, cells were pre-treated with Axitinib at 10 µM for 3 hours before VEGF treatment (n = 3). (c, d, e) Verteporfin (VP) treatment inhibits angiogenesis by MDA-MB-231. MDA-MB-231 were subjected to tube formation assay while being treated with increasing concentrations of VP. Representative images are shown in (c). Scale bar denotes 200 µm. Total tube formation was quantified in (d) and total branch points/field were quantified in (e) (n = 3). (f) Transient knockdown of YAP and/or TAZ in BOEC, MDA-MB231, MCF10A-VEGFR2, HUVEC reduces tube formation but does not cause apoptosis. YAP and/or TAZ were transiently knocked down by siRNA and protein was collected 48 hours after transfection. Cleaved-PARP was measured by western blot. Apoptotic cells that had been treated with 100 nM Taxol for 24 hours were used as a positive control. (g-j) YAP/TAZ are critical for VEGF-induced angiogenesis or vasculogenic mimicry in BOEC (g), MCF10A-VEGFR2 (h), MDA-MB231 (i) and HUVEC (j). Total branch points/field were quantified for tube formation assays using the same images shown in Figure 5. (k) Recombinant CYR61 and ANG2 partially rescue angiogenesis in siYAP/TAZ HUVEC. Total branch points/field were quantified using the same images shown in Figure 5 (n = 3).

Data are represented as mean ± SD.

Supplementary Figure 5

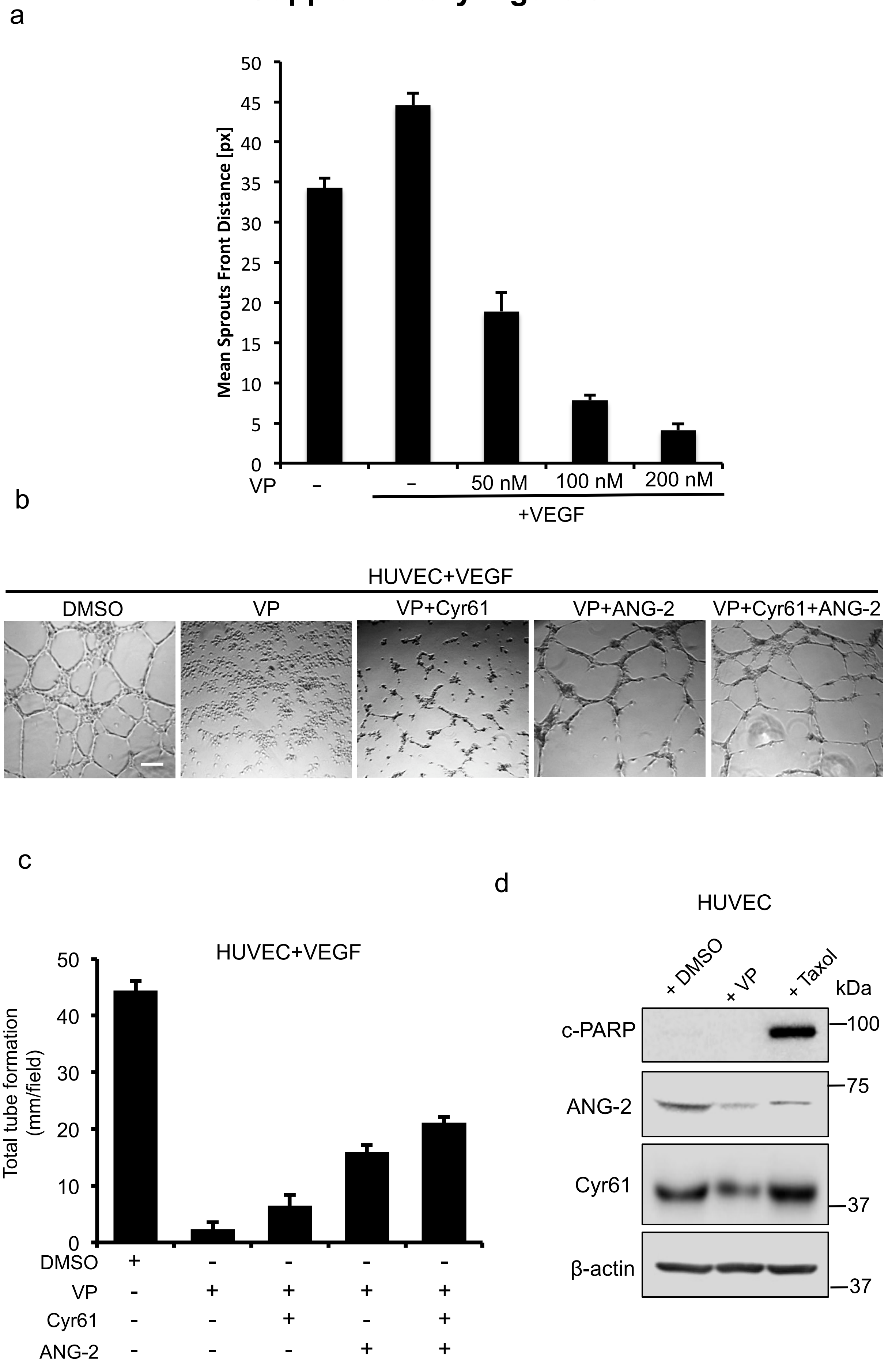

Supplementary Figure 5. Pharmacological inhibition of YAP/TAZ using VP inhibits angiogenesis. (a) Inhibition of YAP and TAZ using VP reduces angiogenesis *ex vivo* in a rat aorta model. Rat aorta were cultured with Matrigel for one week in the presence of the indicated concentration of VP. (b, c) Exogenous CYR61 and ANG-2 can partially rescue tube formation in VP treated HUVEC cells. HUVEC cells were subjected to tube formation assay while being treated with 200 nM concentrations of VP. For some conditions, cells were stimulated with 100 ng mL^{-1} VEGF, 200 ng mL^{-1} CYR61 and/or 200 ng mL^{-1}ANG-2 for the duration of the tube formation assay. Representative images are shown in (b) and quantifications are shown in (c). (d) VP treatment of HUVEC does not cause apoptosis. HUVEC cells were treated with 1 μM VP 24 hour. Cleaved-PARP levels were determined by Western blot. Apoptotic cells that had been treated with 100 nM Taxol for 24 hours were used as a positive control.

REFERENCES

1. Sala-Newby, G. B. & Campbell, A. K. Stepwise removal of the C-terminal 12 amino acids of firefly luciferase results in graded loss of activity. *Biochim. Biophys. Acta* **1206**, 155-160 (1994).

Supplementary Figure 6. Uncropped western blots from primary figures are shown thereafter.

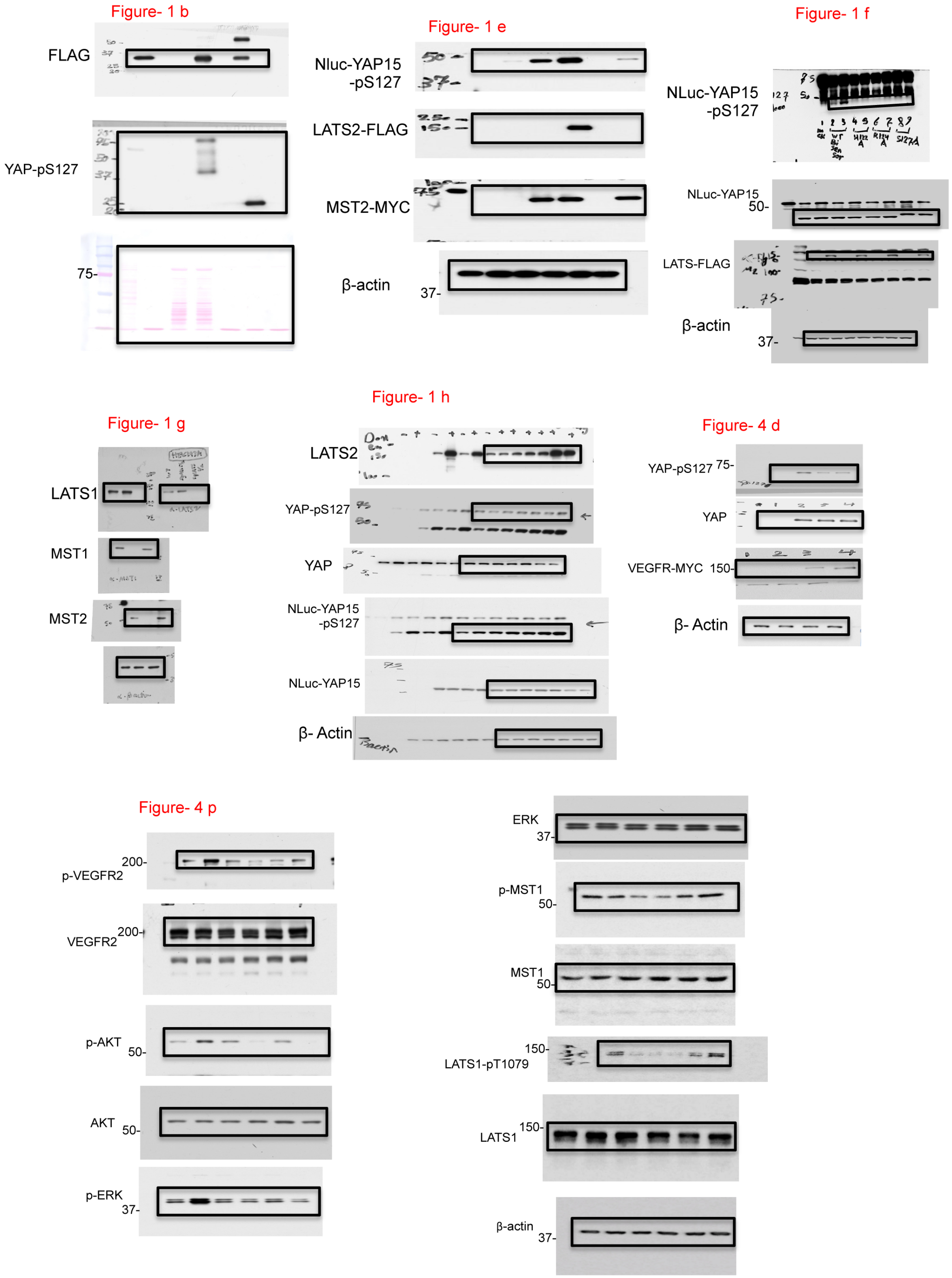

Figure- 5a

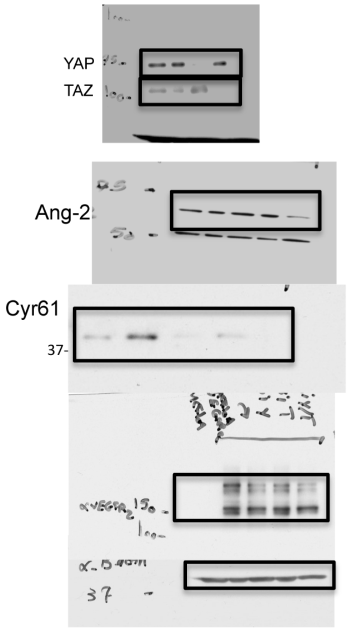

Figure- 5d

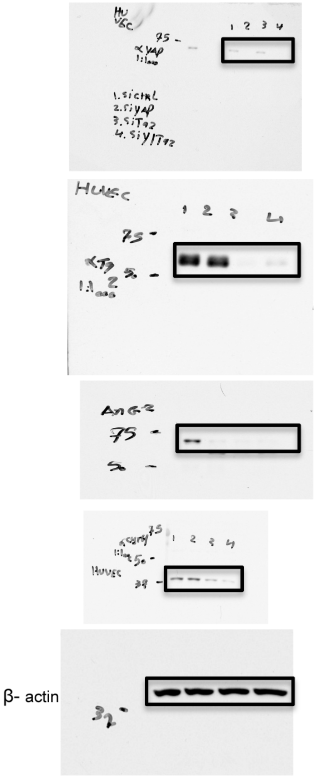

Figure-5 g-

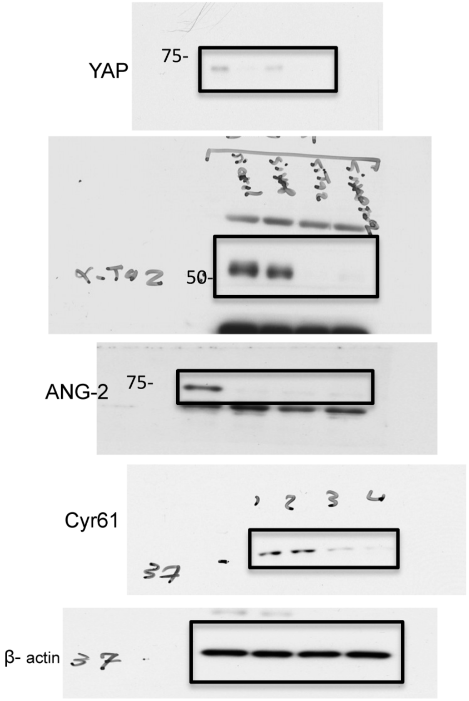

Figure- 5j

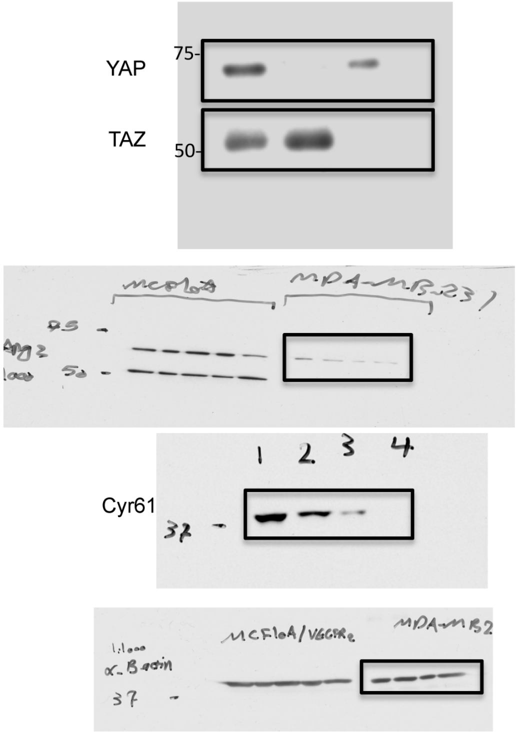

Supplement: Supplementary file 1 — Supplementary Information [file 41467_2018_3278_MOESM1_ESM.pdf]
